# Perioperative blood loss during joint replacement: comparison between patients with and without hemophilia

**DOI:** 10.1186/s13018-022-03217-y

**Published:** 2022-06-21

**Authors:** Shanyou Yuan, Lixia Song, Haoli Jiang, Jinghua Wang, Xianjia Ning, Wenxue Jiang

**Affiliations:** 1 Department of Orthopedics, The Third People’s Hospital of Shenzhen, 29 Bulan Road, Shenzhen, 518112 Guangdong China; 2 Center of Clinical Epidemiology, The Third People’s Hospital of Shenzhen, 29 Bulan Road, Shenzhen, 518112 Guangdong China; 3 Laboratory of Epidemiology, Tianjin Neurological Institute, 154 Anshan Road, Heping District, Tianjin, 300052 China

**Keywords:** Hemophilic arthritis, Total joint replacement, Hemophilia, Blood loss

## Abstract

**Background:**

Although arthroplasty provides satisfactory pain relief, functional improvement, and reduced flexion contracture in patients with hemophilia arthropathy, bleeding remains the primary problem associated with hemophilic arthropathy joint replacement. We aimed to explore the differences in perioperative blood loss (PBL) associated with joint replacement surgery in patients with and without hemophilia.

**Methods:**

This study retrospectively analyzed 61 cases of PBL in patients undergoing joint replacement surgery, including 37 patients with hemophilia and 24 patients without hemophilia. All patients demonstrated severe joint flexion contractures that seriously affected their daily lives and required surgical intervention. PBL was compared between the two groups.

**Results:**

In univariate analysis, the overall (*p* < 0.001) and hidden (*p* < 0.001) blood losses were significantly higher for patients with hemophilia than those for patients without hemophilia. However, after adjustment for multiple factors, there was no significant difference in overall blood loss between the two groups (*p* = 0.731). In addition, sex, age group, and surgical site did not affect blood loss in patients with hemophilia.

**Conclusion:**

Overt bleeding did not increase significantly in patients with hemophilia, compared with that in patients without hemophilia. In terms of blood loss, joint replacement surgery for patients with hemophilia is relatively safe. The results of this study must be verified by a prospective follow-up study with larger sample size.

*Trial registration* Retrospectively registered.

## Background

Hemophilia A and B are rare, congenital, X-linked coagulation disorders caused by factor VIII (FVIII) and factor IX (FIX) deficiencies, respectively [1]. Globally, the number of patients with hemophilia is estimated to be 1,125,000, including 418,000 with severe hemophilia [2]. Hemophilic arthropathy, a serious complication of hemophilia, is characterized by recurring, spontaneous, intra-articular bleeding that causes joint pain and damage to the joint structure [3]. A pathological feature of hemophilic arthritis is repeated intra-articular hemorrhage that causes synovial hyperplasia and inflammation. The proliferative inflammation of the synovial membrane tends to cause blood loss, further aggravating intra-articular hemorrhage and ultimately leading to destruction of the articular cartilage [1]. Evidence suggests that even a single hemorrhage can trigger the inflammatory process, leading to thickening of the synovial membrane and irreversible angiogenesis that results in recurrent bleeding [4].

Treatment of mild to moderate hemophilic arthritis may include support/splinting, joint movement, progressive strengthening, stretching, and pain management styles, as well as patient education on pain management, activities to avoid, and other joint disease management recommendations [5]. For patients suffering from intractable pain and/or lifestyle-limiting range of motion defects, invasive interventions can be provided, including corticosteroid injections, viscosupplementation, synovectomy [6, 7], joint debridement, arthrodesis, and sliding membranectomy accompanied by resection of the radial head [6].

A previous study attempted to search for biomarkers that could be used to monitor bleeding and assess the progression of hemophilic arthropathy in clinical practice and evaluate the effectiveness of treatments. Nevertheless, the clinical use of biomarkers was still limited in clinical practice because of a lack of standardization [8, 9]. Thus, total joint replacement may be offered to patients with excessive pain or loss of range of motion and function and who are unresponsive to conservative treatment.

Although previous studies reported that arthroplasty provides satisfactory pain relief, functional improvement, and reduced flexion contracture in patients with hemophilia arthropathy [10, 11], bleeding remains the primary problem associated with hemophilic arthropathy joint replacement. Studies regarding PBL associated with joint replacements in patients with and without hemophilia are lacking. Therefore, this study investigated the differences in PBL associated with joint replacement in patients with and without hemophilia.

## Methods

### Participants

We retrospectively analyzed 61 patients with hemophilia A, including 46 men and 15 women. Among the patients, 34 had hip joint disease, and 17 had knee joint disease. Preoperative imaging showed that the damaged joints were all severely deformed; bone degeneration was evident. According to Arnold and Hilgartner's staging [3], all cases of hemophilia arthritis were Stage V (end-stage).

Preoperatively, the patients with hemophilia underwent routine coagulation factor pharmacokinetic assessments and received human recombinant coagulation factor VIII. The blood concentration of the coagulation factor was measured immediately and 30 min, 9 h, 24 h, and 48 h after its infusion. These data were used to determine the half-life of the coagulation factor in the body while also testing for the presence of coagulation factor inhibitors. Regular infusion of coagulation factor VIII during the postoperative period ensured that the coagulation factor concentration remained at 100% that on the day of surgery and 3 days after surgery. No patients used heparin prophylactically, and tranexamic acid was only for topical use on wounds. The coagulation function of patients without hemophilia was normal.

### Information collection and definitions

Clinical information (presenting signs and symptoms) and demographic information for the patients were obtained from the hospital’s computerized medical record information system. The main symptoms, recorded by the doctor-in-charge, were based on each patient’s performance and self-reporting.

The demographic information included each patient’s sex and age. The participants were grouped into three age categories: < 40 years, 40–64 years, and >  64 years. Each patient’s body mass index (BMI) was calculated as the individual’s weight (kg) divided by the square of the individual’s height (m^2^) [12].

### PBL evaluation

The preoperative blood volume was calculated using the Nadler equation [13]. Data were collected regarding intraoperative blood loss, blood transfusions, and 24-h postoperative wound drainage. The PBL was calculated according to the Gross equation [14], and the theoretical total blood loss (TBL) was calculated according to the serum hematocrit (HCT).

The following equation was used: theoretical TBL = preoperative blood volume (BV) × (preoperative HCT – postoperative HCT)/mean HCT, where BV (L) = K1 × height (m^3^) + K2 × weight (kg) + K3. For males, variables are defined as follows: K1 = 0.3669, K2 = 0.03219, and K3 = 0.6041. For females, variables are defined as follows: K1 = 0.3561, K2 = 0.03308, and K3 = 0.1833.

TBL was calculated as the theoretical TBL plus total blood transfusion volume. Dominant blood loss (DBL) was calculated as the intraoperative blood loss plus PBL, and hidden blood loss (HBL) was calculated as TBL minus DBL.

### Statistical analyses

Continuous variables (age, BMI, hematocrit [HCT], blood loss) are presented as means and standard deviations (SDs). Comparisons of the continuous variables were made using Student’s *t*-tests. Categorical variables (age, group, and surgical site) were presented as numbers and frequencies; between-group comparisons were performed using χ^2^ tests. All analyses were conducted using SPSS for Windows (version 22.0; SPSS; Chicago, IL, USA); *p* < 0.05 was considered statistically significant.

## Results

This study included 46 men (75.4%) and 15 women (24.6%). The mean age of the patients was 51.9 years, and the 40–65 age group accounted for the largest proportion (54.1%). Total hip replacements accounted for 54.1% of the surgeries. The mean preoperative and intraoperative HCTs were 40% and 30%, respectively (Table 1).

**Table 1:** Characteristics of participants in this study

Category	Men	Women	Total
Total:	46	15	61
Age, means (SD), years	46.8 (13.5)	67.7 (19.1)	51.9 (17.4)
*Age group, n (%)*			
< 40 years	14 (30.4)	1 (6.7)	15 (24.6)
40 ~ 64 years	27 (58.7)	6 (40.0)	33 (54.1)
> 64 years	5 (10.9)	8 (53.3)	13 (21.3)
*Hemophilia, n (%)*			
Yes	36 (78.3)	1 (6.7)	37 (60.7)
No	10 (21.7)	14 (93.3)	24 (39.3)
*Surgical site, n (%)*			
THR	18 (39.1)	15 (100)	33 (54.1)
BHR	1 (2.2)	0	1 (1.6)
TKR	27 (58.7)	0	27 (44.3)
BMI, means (SD), Kg/m^2^	23.7 (3.6)	21.4 (2.9)	23.1(3.6)
*HCT, %*			
Preoperative	0.4 (0.1)	0.3 (0.1)	0.4 (0.1)
Intraoperative	0.3 (0.1)	0.3 (0.1)	0.3 (0.1)
Apparent blood loss	0.4 (0.4)	0.6 (0.5)	0.5 (0.4)
Hidden blood loss	1.4 (0.9)	0.8 (0.7)	1.3 (0.9)
Blood transfusion	0.1 (0.2)	0.4 (0.3)	0.2 (0.3)

The mean preoperative HCT of the patients with hemophilia (0.42%) was significantly higher than that of the patients without hemophilia (0.37%, *p* = 0.002). Overall blood loss (*p* < 0.001) and HBL (*p* < 0.001) of patients with hemophilia were significantly higher than those of patients without hemophilia. However, a significant between-group difference in the apparent intraoperative blood loss was not observed (*p* = 0.731). In this study, the rate of red blood cell transfusion in patients without hemophilia was higher than that in patients with hemophilia (41.7% vs. 21.6%, respectively), but there was no significant difference (*p* = 0.094) (Table 2).

**Table 2:** The difference in bleeding volume and RBC transfusion between hemophilia patients and non-hemophilia patients

Category	Hemophilia		*p*
	Yes	No	
*HCT,%*			
Preoperative	0.42 (0.05)	0.37 (0.07)	0.002
Intraoperative	0.29 (0.07)	0.29 (0.06)	0.779
Total blood loss	1.95 (1.07)	1.01 (0.51)	< 0.001
Apparent blood loss	0.45 (0.43)	0.49 (0.38)	0.731
Hidden blood loss	1.62 (0.96)	0.72 (0.52)	< 0.001
Blood transfusion	0.13 (0.26)	0.21 (0.28)	0.228
RBC transfusions, *n* (%)	8 (21.6)	10 (41.7)	0.094

Table 3 shows that there were no significant differences in TBL, HBL, or DBL based on sex, age group, or surgical site (all, *p* > 0.05).

**Table 3:** Influencing factors of blood loss among hemophilia patients

Category	Blood loss (L)					
	Total	P	Apparent	P	Hidden	P
Gender:		0.798		0.555		0.210
Men	1.94 (1.08)		0.46 (0.43)		1.59 (0.95)	
Women	2.22 (0)		0.20 (0)		2.82 (0)	
Age group:		0.206		0.599		0.253
< 40 years	2.27 (1.03)		0.51 (0.59)		1.88 (0.95)	
40 ~ 64 years	1.79 (1.07)		0.43 (0.33)		1.49 (0.96)	
> 64 years	–		–		–	
Surgical site:		0.722		0.567		0.285
THR	2.05 (1.13)		0.39 (0.35)		1.90 (1.06)	
TKR	1.91 (1.07)		0.48 (0.46)		1.51 (0.92)	

## Discussion

There was no significant difference in the amounts of apparent bleeding between patients with and without hemophilia, but the HBL and overall blood loss volumes were significantly higher in patients with hemophilia than those in patients without hemophilia.

Joint replacement in patients with hemophilia includes obstacles, particularly intraoperative and postoperative bleeding caused by abnormal coagulation function. Previous studies have shown that effective clotting factor replacement can reduce the risk of perioperative bleeding for patients undergoing total knee arthroplasty. Macgillivray et al. [15] showed that the postoperative drainage volume for patients not treated with antifibrinolytic drugs during the first stage of bilateral total knee arthroplasties was 918 mL. Additionally, Heeg et al. [16] reported that 9 patients (12 knees) with hemophilia had an average blood loss of 1100 mL (300–1200 mL) after complete hemostasis during surgery. In this study, the TBL volume for patients with hemophilia was 1.95 L, which was significantly higher than that for patients without hemophilia (1.01 L). However, there was no significant difference in apparent blood loss between the groups of patients. In another study, total knee replacement was an effective surgical procedure in patients with hemophilia, although the long-term and early surgical outcomes (including postoperative fibrosis, previous synovectomy, and presence of inhibitors) were slightly suboptimal in patients with hemophilia, compared with those in patients without hemophilia [17]. The results of the present study indicate that joint replacement in patients with hemophilia is safe, provided there is a strict control of coagulation indicators, and close monitoring of postoperative HCT is also necessary for patients with hemophilia.

However, the factors affecting blood loss remain controversial [18]. In patients with hemophilic knee arthritis, the blood loss during total knee arthroplasty varies from 300 to 3000 mL [19]. Studies have shown that the amount of blood lost during total knee arthroplasty in these patients is related to HCT and average red blood cell density [18, 20]. This relationship exists because HCT can affect platelet adhesion to the damaged endothelium, possibly affecting intraoperative blood loss. In patients with an HCT of < 38.5%, the average intraoperative blood loss was observed to be 1000 mL; in patients with an HCT of > 38.5% (a parameter that depends on red blood cell density), the intraoperative blood loss was higher in patients with increased red blood cell density.

With careful perioperative preparation and the help of FVIII and other blood products, hemophilia is no longer an absolute contraindication to surgery. Symptomatic and supportive treatment, anti-infective treatment, and joint replacement in patients with hemophilia are feasible and worthwhile. This paper provides a certain reference for clinical practice, but because the number of included cases is small, more clinical data are still needed to verify the results.

This study has some limitations. First, the patients in this study were all treated at a single center, and the represented population was limited. Second, this was a retrospective study that involved a small number of cases. Thus, a prospective, controlled study involving a larger number of patients is necessary to confirm the present results. Finally, this study did not collect information regarding patient medication use and medical history. A prospective follow-up study will need to collect additional relevant information and involve a multivariate analysis.

## Conclusion

The amount of bleeding in patients with hemophilia did not increase significantly during joint replacement surgery, compared with that in patients without hemophilia. This study shows that joint replacement surgery is generally safe for patients with hemophilia, provided appropriate monitoring and follow-up are included in their care.

## Data Availability

The datasets generated and/or analyzed during the current study are available from the corresponding author upon reasonable request.

## Abbreviations

FVIII: Factor VIII
FIX: Factor IX
BMI: Body mass index
HCT: Hematocrit
SDs: Standard deviations
DBL: Dominant blood loss
HBL: Hidden blood loss
PBL: Perioperative blood loss
TBL: Total blood loss

## References

[1] Mannucci PM, Tuddenham EG (2001). The hemophilias–from royal genes to gene therapy. N Engl J Med.

[2] Iorio A, Stonebraker JS, Chambost H, Makris M, Coffin D, Herr C (2019). Establishing the prevalence and prevalence at birth of hemophilia in males: a meta-analytic approach using national registries. Ann Intern Med.

[3] Arnold WD, Hilgartner MW (1977). Hemophilic arthropathy. Current concepts of pathogenesis and management. J Bone Joint Surg Am..

[4] van Vulpen LF, van Meegeren ME, Roosendaal G, Jansen NW, van Laar JM, Schutgens RE (2015). Biochemical markers of joint tissue damage increase shortly after a joint bleed; an explorative human and canine in vivo study. Osteoarthr Cartil.

[5] Marshall Brooks M, Tobase P, Karp S, Francis D, Fogarty PF (2011). Outcomes in total elbow arthroplasty in patients with haemophilia at the University of California, San Francisco: a retrospective review. Haemophilia.

[6] Silva M, Luck JV (2007). Radial head excision and synovectomy in patients with hemophilia. J Bone Joint Surg Am.

[7] Siegel HJ, Luck JV, Siegel ME, Quinones C (2001). Phosphate-32 colloid radiosynovectomy in hemophilia: outcome of 125 procedures. Clin Orthop Relat Res.

[8] Pasta G, Annunziata S, Polizzi A, Caliogna L, Jannelli E, Minen A (2020). The progression of hemophilic arthropathy: the role of biomarkers. Int J Mol Sci.

[9] Pasta G, Jannelli E, Ivone A, et al. The role of six biomarkers in diagnosis of hemophilic arthropathy: review of the literature. J Biol Regul Homeost Agents. 2020. 34 (3 Suppl. 2): 7–13. Advances in musculoskeletal diseases and infections - Sotimi 2019. 32856434

[10] Song SJ, Bae JK, Park CH, Yoo MC, Bae DK, Kim KI (2018). Mid-term outcomes and complications of total knee arthroplasty in haemophilic arthropathy: a review of consecutive 131 knees between 2006 and 2015 in a single institute. Haemophilia.

[11] Ernstbrunner L, Hingsammer A, Imam MA, Sutter R, Brand B, Meyer DC (2018). Long-term results of total elbow arthroplasty in patients with hemophilia. J Shoulder Elbow Surg.

[12] Wang J, Taylor AW, Zhang T, Appleton S, Shi Z (2018). Association between body mass index and all-cause mortality among oldest old Chinese. J Nutr Health Aging.

[13] Nadler SB, Hidalgo JH, Bloch T (1962). Prediction of blood volume in normal human adults. Surgery.

[14] Gross JB (1983). Estimating allowable blood loss: corrected for dilution. Anesthesiology.

[15] MacGillivray RG, Tarabichi SB, Hawari MF, Raoof NT (2011). Tranexamic acid to reduce blood loss after bilateral total knee arthroplasty: a prospective, randomized double blind study. J Arthroplasty.

[16] Heeg M, Meyer K, Smid WM, Van Horn JR, Van der Meer J (1998). Total knee and hip arthroplasty in haemophilic patients. Haemophilia.

[17] Pasta G, Vanelli R, Jannelli E (2018). Primary total knee replacement in hemophiliacs: experience of a single institution over fourteen years of surgical procedures. J Biol Regul Homeost Agents.

[18] Hu Y, Li Q, Wei BG, Zhang XS, Torsha TT, Xiao J (2018). Blood loss of total knee arthroplasty in osteoarthritis: an analysis of influential factors. J Orthop Surg Res.

[19] Shurkhina ES, Polyanskaya TY, Zorenko VY, Azimova MK, Nesterenko VM, Ataullakhanov FI (2016). Effect of hematocrit and erythrocyte density on intraoperative blood loss in hemophilia a patients during total knee arthroplasty. Bull Exp Biol Med..

[20] Kasper CK, Boylen AL, Ewing NP, Luck JV, Dietrich SL (1985). Hematologic management of hemophilia A for surgery. JAMA.

